# Complications of enterostomy and related risk factor analysis of very early onset inflammatory bowel disease with interleukin-10 signalling deficiency: a single-centre retrospective analysis

**DOI:** 10.1186/s12876-020-1160-4

**Published:** 2020-01-13

**Authors:** Zifei Tang, Zhiheng Huang, Weili Yan, Yi Zhang, Peng Shi, Kuiran Dong, Ying Gong, Min Ji, Yuhuan Wang, Zhuowe Yu, Ying Huang

**Affiliations:** 1 Department of Gastroenterology, Children’s Hospital of Fudan University, 399 Wanyuan Road, Minhang District, Shanghai, 201102 People’s Republic of China; 2 Department of Clinical Epidemiology, Children’s Hospital of Fudan University, Shanghai, 201102 China; 3 Department of Information, Children’s Hospital of Fudan University, Shanghai, 201102 China; 4 Department of Surgery, Children’s Hospital of Fudan University, Shanghai, 201102 China; 5 Department of Radiology, Children’s Hospital of Fudan University, Shanghai, 201102 China

**Keywords:** IL10/IL10R, Very early onset inflammatory bowel disease, Enterotomy

## Abstract

**Background:**

Interleukin-10 (IL10) signalling pathway deficiency results in severe very early onset inflammatory bowel disease (VEOIBD), and enterostomy is often inevitable. However, studies in these surgical populations are lacking. This study aims to determine the enterostomy characteristics, postoperative complications and related risk factors in enterostomy patients.

**Methods:**

From March 1, 2015, to December 31, 2018, patients with IL10R-mutation who underwent enterostomy were recruited for analysis. We collected data on the patients’ clinical characteristics, enterostomy characteristics, postoperative complications and related risk factors.

**Results:**

Twelve patients required emergency enterostomy, and 10 patients underwent elective enterostomy. Twelve patients experienced postoperative complications, including wound infection (27.3%), wound dehiscence (18.2%), reoperation (18.2%), etc. Compared with the pre-enterostomy values, there was a decrease in C-reactive protein (CRP) (*P* = 0.001), an increase in albumin (*P* = 0.001) and an improvement in the weight-for-age (*P* = 0.029) and body mass index (BMI) Z-scores (*P* = 0.004) after enterostomy. There was a significant difference between the pre-operation and postoperation medicine expenses (*P* = 0.002). Univariate binary logistic regression analysis revealed a statistically significant influence of CRP (OR: 1.43, 95% CI: 1.07–1.91, *P* = 0.016) and a tendency towards a significant influence of intestinal perforation, albumin level, BMI Z-score and weighted paediatric Crohn’s disease activity index (wPCDAI). Multivariate logistic regression analysis showed that CRP (OR: 1.40), wPCDAI (OR: 2.88) and perforation (OR: 1.72) showed a tendency to behave as independent risk factors for postoperative complications, but the results were not significant (all *P* > 0.05).

**Conclusions:**

Surgery and enterostomy showed benefits for VEOIBD with IL-10 signalling deficiency. The timing of intervention, potential postoperative complications, economic burden and other related problems should be considered.

## Background

Very early onset inflammatory bowel disease (VEOIBD) in children under the age of 6 years diagnosed with IBD is often associated with a monogenetic disorder [1]. More than 60 genes associated with VEOIBD have been identified through next-generation sequencing and genetic linkage analysis [2, 3].

Interleukin-10 (IL10)/interleukin-10 receptor (IL10R) mutations have been identified in a subset of infants with severe VEOIBD. Our group studied a large cohort of patients with VEOIBD caused by IL-10R defects [4, 5]. This type of VEOIBD often presents as perianal diseases and intestinal ulcers, perforation, and obstruction; VEOIBD responds poorly to medical therapies and requires early surgical interventions [4, 6]. Elective surgery and emergency surgery are often performed in children with severe gastroenterological symptoms [7].

However, descriptions of enterostomy in VEOIBD patients, especially those with IL10R gene mutations, are scarce. This retrospective study aims to describe the characteristics of VEOIBD patients with IL10R mutation after enterostomy, enterostomy complications and related risk factors.

## Methods

### Patients

This study was approved by the Ethics Committee of the Children’s Hospital of Fudan University. From March 1, 2015, to December 31, 2018, 25 VEOIBD patients with IL10R mutation who underwent enterostomy were recruited. Three patients were excluded because enterostomy was performed outside of the hospital or they lacked complete data.

Data were collected from the electronic medical records of the patients. Follow-up information, including age of onset, presence of perianal lesions, surgical procedures, clinical outcomes, family history, gene information, etc. were also collected. The weighted paediatric Crohn’s disease activity index (wPCDAI) was used to assess the clinical course of the disease.

### Classification of complications

Complications of enterostomy for VEOIBD patients with IL10R mutation were noted. Complications included haemorrhage, wound infection, seepage, incisional hernia reoperation, death, etc.

### Nutritional evaluation

For the included patients, the available height, weight, weight-for-age Z-score, height-for-weight Z-score, and BMI Z-score at the time of first diagnosis were determined using the World Health Organization (WHO) Anthro software (version 3.2.2) [8]. The 2015 consensus on paediatric malnutrition guidelines was used to categorize BMI-for-age Z-scores into the following levels of malnutrition: none, > − 1; mild, − 1 to − 1.9; moderate, − 2 to − 2.9; and severe, <− 3 [9]. C-reactive protein (CRP) and albumin levels were also collected for the nutritional evaluation.

### Analysis

Data were analysed using SPSS 24.0 for Windows (SPSS Inc., Chicago, IL, USA). Continuous data were presented as the mean and standard deviation (SD) or median and interquartile range (IQR). Categorical variables were reported as the frequency and percentage. Comparisons between groups (CRP, RMB/month discharge, weight-for-age Z-score, height-for-weight Z-score, and BMI Z-score) were made by using the Mann-Whitney U test for non-normally distributed data; Student’s t-test was used for albumin comparison because the data were normally distributed. A two-tailed *P* value < 0.05 was considered statistically significant. A multivariate analysis was performed using binary logistic regression to determine the independent risk factors associated with enterostomy complications. Because the traditional level of 0.05 might fail to identify variables known to be important, for inclusion in the multivariate mode, we chose only factors that had *P* values < 0.1 in the univariate analysis for inclusion in the multivariate analysis [10].

## Results

### Baseline characteristics of the patients who underwent enterostomy

From March 1, 2015, to December 31, 2018, a total of 25 patients underwent enterostomy (14 males and 11 females). Of these patients, 22 had complete data and were enrolled. Twenty-one patients presented IL10RA gene mutations and the mutation sites of 14 patients were previously reported [4, 5, 11]. One patient presented novo homozygous mutations of the IL10RB gene (c.612G > C, p. W204C). The variant pathogenic analysis is shown in Additional file 1: Table S1.

Throughout the study period, the medical treatments included haematopoietic stem cell transplantation (HSCT) (45.5%), mesalamine (45.5%), thalidomide (77.3%), infliximab (13.6%), azathioprine (4.5%), and faecal microbiota transplant (FMT) (4.0%). As of the last follow-up, none of the patients had undergone repeated HSCT. The basal data are shown in Table 1.

**Table 1:** Demographic characteristics of children with VEOIBD with IL10R deficiency

Characteristic	
Male: *n* (%)	14(63.6%)
Age at diagnosis (m): median [IQR]	9(3.5,21.5)
Duration of follow-up (m): median [IQR]	16.0(4.8,31.3)
Diarrhoea: *n* (%)	21(94.5)
Fever: *n* (%)	11(50.0%)
Oral ulcer: *n* (%)	7(31.8%)
Perianal disease: *n* (%)	16(72.7%)
Growth parameters at first hospitalization	
Weight-for-age Z-score: median [IQR]	−3.5(−4.7, −2.7)
Height-for-age Z-score: median [IQR]	−3.1(− 4.7, − 2.2)
BMI Z-score: median [IQR]	−2.6(− 3.8, − 0.53)
Medication usage prior to enterostomy	
Thalidomide: *n* (%)	17(77.3%)
HSCT: *n* (%)	10(45.5%)
Mesalazine: *n* (%)	10(45.5%)
Infliximab: *n* (%)	3(13.6%)

### Characteristics at the time of first surgery

For the included patients, emergency or selective surgeries were employed for different conditions. Twelve patients underwent emergency surgeries (6 for intestinal obstruction and 6 for intestinal perforation). Ten patients underwent elective surgeries because of refractory intestinal infection (7 cases), intestinal stenosis (2 cases) and severe perianal diseases (1 case) and after medical treatment. The median and IQR age of first surgery was 12.0 months [5.0, 27.3]. The type of surgery is shown in Table 2. Among the enterostomies, ileostomy was the most common surgery, followed by jejunostomy.

**Table 2:** Enterotomy information of children with VEOIBD with IL10R deficiency

Operation-related information	
Pre-operative assessment	
Abdominal distension: *n* (%)	12(54.5%)
Malnutrition (%)	8(36.4%)
Intestinal obstruction on X-ray examination: *n* (%)	7(22.7%)
Intestinal perforation on X-ray examination: *n* (%)	6(22.7%)
CRP (mg/L)	70.5(41.3153.8)
WBC (*10^9/L)	11.5(8.9,14.6)
Hb (g/L)	92.6(85.0,103.8)
Albumin (g/L)	26.59 ± 6.43
wPCDAI	58.9 ± 17.9
Operation opportunity and type	
Emergency surgery: *n* (%)	12(54.5%)
Ileostomy: *n* (%)	13(59.1%)
Jejunostomy: *n* (%)	8(36.4%)
Transversostomy: *n* (%)	1(4.5%)
Findings during operation	
Perforation in small bowel: *n* (%)	2(9.1%)
Perforation in ileocaecal junction	2(9.1%)
Perforation in colon and rectum	7(31.8%)
Peritonitis: *n* (%)	6(27.3%)
Intestinal adhesion or dilatation: *n* (%)	12(54.5%)
Enterostomy complications	
Wound infection: *n* (%)	6(27.3%)
Wound dehiscence: *n* (%)	4(18.2%)
Wound bleeding: *n* (%)	2(9.1%)
Reoperation: *n* (%)	6(27.3%)
Death: *n* (%)	2(9.1%)
Short bowel syndrome: *n* (%)	2(9.1%)
Parastomal hernias: *n* (%)	1(4.5%)
Prognosis (death): *n* (%)	6(27.3%)

**Fig. 1:**
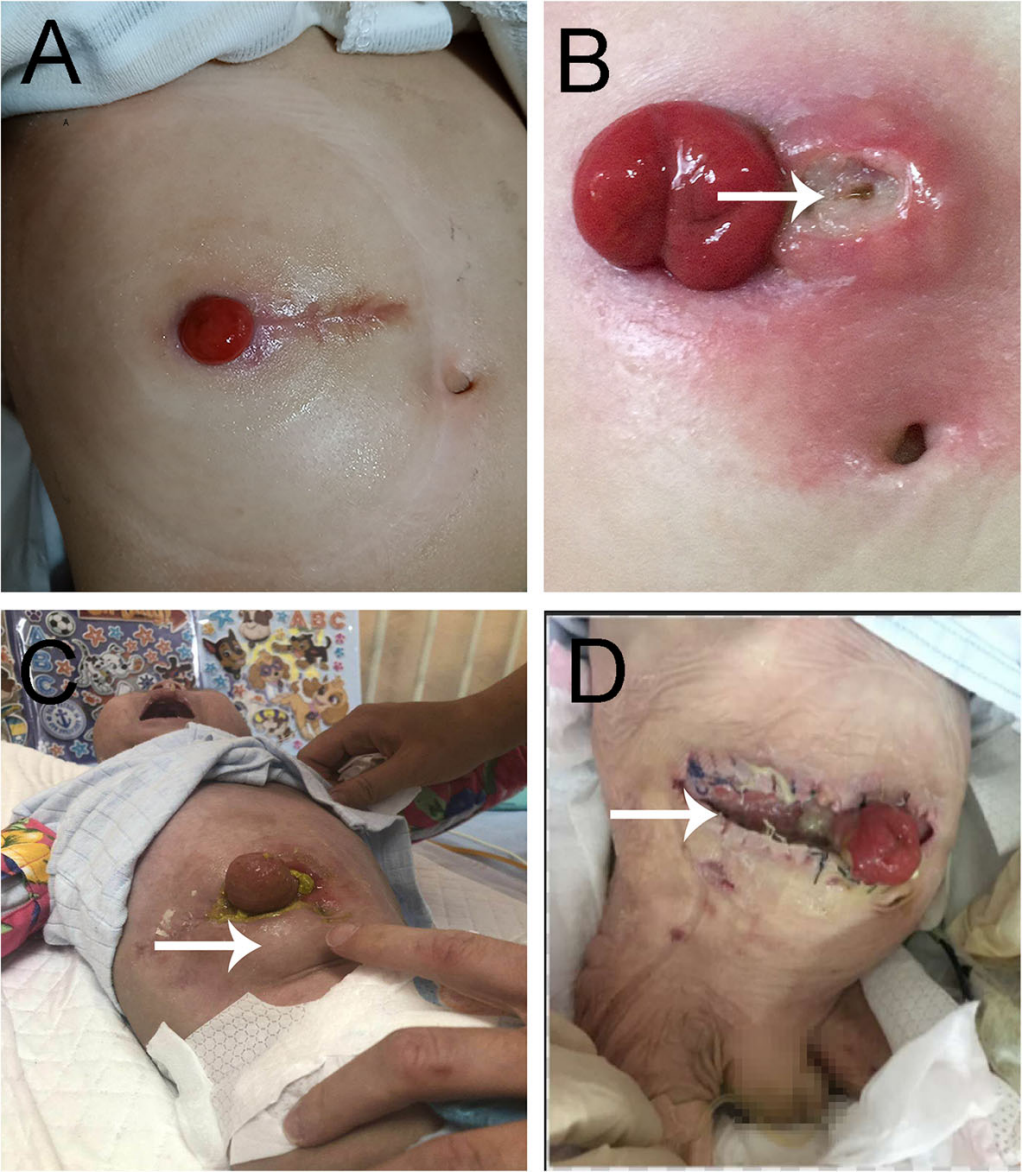
Enterotomy characteristics in IL10R-mutation VEOIBD patients with and without complications. **a** Enterostomy without complication; **b** Wound infection; **c** Parastomal hernias; **d** Wound dehiscence

Complications of enterostomy occurred in some of the patients (Table 2). The most common postoperative complication was wound infection (27.3%), followed by wound dehiscence (18.2%), reoperation (18.2%), death (9.1%), short bowel syndrome (9.1%), wound bleeding (9.1%) and parastomal hernia (4.5%) (Fig. 1).

### Comparison of pre-operation and postoperation data

Pre-operation and 14-day postoperation laboratory data were collected. After the operation, the CRP level decreased dramatically with increasing albumin (*P* = 0.001). Growth parameters at two time points (at the first hospitalization and on December 31, 2018) were recorded. The weight-for-age Z-score and BMI Z-score improved significantly after surgery (*P* = 0.029 and 0.004, respectively).

**Fig. 2:**
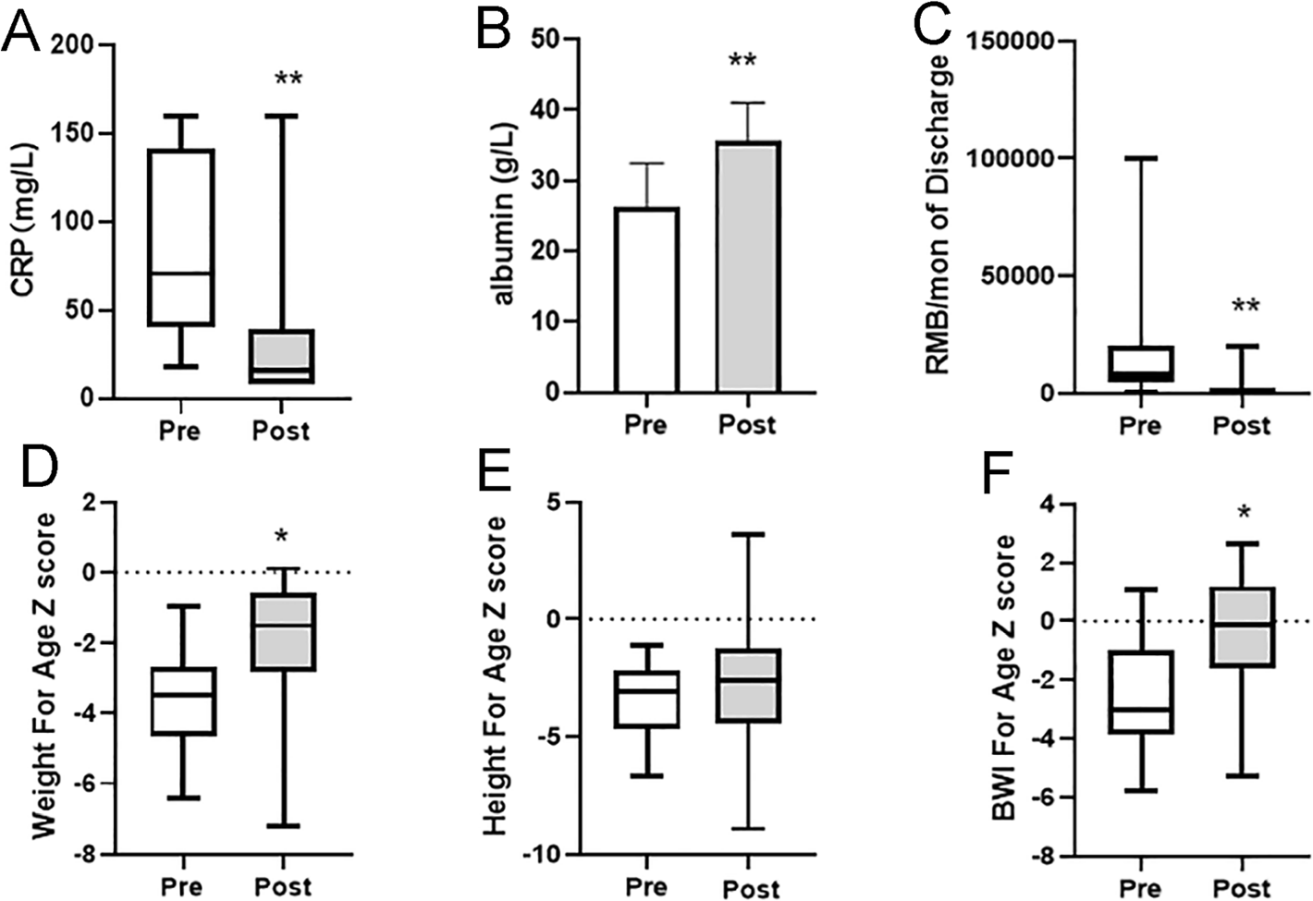
Comparison of clinical variables before and after enterostomy. **a** Pre-operation and 14-day postoperation CRP levels. **b** Pre-operation and 14-day postoperation albumin levels. **c** Comparison of pre- and postoperation medicine expenses (RMB per month). **d** Weight-for-age Z-scores at the first admission and last follow-up. **e** Height-for-age Z-scores at the first admission and last follow-up. **f** BMI-for-age Z-scores at the first admission and last follow-up. *: ***p*** < 0.05, **: ***p*** < 0.01

No difference was observed in the total patient discharge expenses pre- and postoperation (median and IQR: 9000 [4250, 22,500] RMB/month vs. 3200 [2075, 8500] RMB/month, *P* = 0.068) because of the additional expense of enterostomy care (Fig. 2). However, there was a significant difference in the pre- and postoperation expenses for medicine (median and IQR:9000 [4250, 22,500] RMB/month vs. 500 [350, 2625] RMB/ month, *P* = 0.003).

### Risk factors for enterostomy complications

The risk factors were analysed for two groups: those with and without enterostomy complications. We found that there was a significant difference in CRP levels (OR 1.413, 95% CI: 1.069, 1.914, *P* = 0.016) and a tendency towards significant differences in intestinal perforation (OR 4.667, 95% CI: 0.765, 28.466, *P* = 0.095), albumin level (OR 0.083, 95% CI: 0.693, 1.002, *P* = 0.053), BMI Z-score (OR 0.076, 95% CI: 0.480, 1.036, *P* = 0.075) and wPCDAI (OR 3.338 95% CI: 0.850,13.515, *P* = 0.084).

Variables with statistical significance in the univariate analysis were considered in the multivariate logistic regression analysis. CRP level, intestinal perforation, albumin level, BMI Z-score and wPCDAI were included in the independent risk factor analysis. Although no significant differences were observed after the multivariate analysis, CRP level (OR1.400, *P* = 0.195), wPCDAI (OR 2.881, *P* = 0.260) and intestinal perforation (OR1.72, *P* = 0.737) had a tendency towards being independent risk factors (Table 3).

**Table 3:** Risk factor analysis for enterotomy complications of children with VEOIBD with IL10R deficiency

	Univariate analysis		Multivariate analysis	
	OR (95% CI)	*P*	OR (95% CI)	*P*
Gender	4.0(0.59, 27.25)	0.157		
Age of enterotomy	0.94(0.87, 1.01)	0.100		
Type of surgery	0.71(0.13, 3.87)	0.696		
CRP (mg/L)	1.43(1.07, 1.91)	0.016	1.40(0.84, 2.33)	0.195
X-ray (obstruction/ perforation)	2.16(0.71, 6.62)	0.177		
Positive abdominal sign	3.00(0.53, 17.16)	0.217		
Perforation	4.67(0.77, 28.47)	0.095	1.72(0.07, 40.33)	0.737
Albumin (g/L)	0.83(0.69, 1.00)	0.053	1.00(0.75, 1.34)	0.999
Anaemia (g/L)	1.01(0.93, 1.09)	0.839		
BMI Z-score	0.71(0.48, 1.04)	0.075	1.03(0.59, 1.79)	0.930
Simplified endoscopic score (SES)	1.09(0.92, 1.30)	0.328		
wPCDAI	3.39(0.85, 13.52)	0.084	2.881(0.457, 18.150)	0.260

### Follow-up

At the last follow-up, sixteen patients were still alive, and six patients had died. In our study, 3 patients received secondary stoma construction apart from the primary incision. Two patients underwent reoperation for adhesive intestinal obstruction or prolapse.

Ten patients received HSCT after enterostomy; the median and IQR for the time interval between enterotomy and HSCT were 120.0 days [80.3237.3]. Of the patients who received HSCT after enterostomy, nine experienced confirmed successful immune reconstitution and symptom remission, and one patient died of graft failure and sepsis. Three patients experienced remission after enterostomy with no need for HSCT. The remaining patients had improved growth parameters after enterostomy and were waiting for HSCT at the end of the study. Till the time of this article draft, the rate of enterostomy closure was 9.1% (2/22), and the time interval was 17.5 months for one patient and 25.8 months for another.

## Discussion

VEOIBD, especially with IL10/IL10R signalling pathway deficiency, is characterized by severe enterocolitis, perianal disease and proctitis involving the colon and terminal ileum [12]. The lesions associated with IL10 are distributed from the jejunum to the anorectum, and the terminal ileum and colon are the most frequently involved sites. Stricture, perforation, abscess, fistula, and severe perianal disease are common complications in IL10R-mutation IBD patients. Surgery is indicated after medical treatment is unsuccessful or after disease complications develop [13, 14]. However, published data on the complications, possible risk factors and other postoperative outcomes in IL10R-mutation IBD patients are scarce.

For paediatric IBD patients, the benefits of enterostomy include alleviation of disease severity, extended lifespan and reduced risk of death [14–16]. Although surgery is never curative for paediatric IBD patients, it has the ability to transform the disease process. For monogenic VEOIBD, timely enterostomy may offer opportunities for further HSCT [17]. In our study, 22 IL10R-mutation patients underwent enterostomy, and 12 patients underwent an emergency operation because of intestinal obstruction or perforation. Surgical procedures such as ileostomy and colostomy can induce remission of the disease.

Fahy et al. reported that in paediatric patients with severe refractory Crohn’s colitis, total colectomy offers an opportunity to improve nutrition and growth and reduce steroid use with a reasonable likelihood of restoring intestinal continuity [18]. Maxwell et al. reported that in paediatric patients with refractory colonic IBD, a diverting ileostomy can be a successful intervention to induce clinical stability [19]. In our study, the surgical approach allowed patients to decrease the use of metronidazole, thalidomide and other drugs. The patients experienced disease remission and developmental growth after enterostomy, with an increase in albumin levels, weight for age Z-scores and BMI Z-scores. Furthermore, 7 patients gained the opportunity to undergo HSCT, and 6 patients presented successful immune reconstitution and symptom remission after enterostomy.

For severe VEOIBD with IL10 gene mutations, appropriate surgical treatments should be used. Emergency operation indications, such as intestinal perforation, complete obstruction, or generalized peritonitis, may occur. For medically resistant patients or those with severe perianal disease, elective surgeries should be considered [15]. Leeds et al. reported that delayed operations for acute ulcerative colitis were associated with increased postoperative complications, increased lengths of stay, and increased hospital costs. Therefore, immediate surgical intervention for medically refractory ulcerative colitis is suggested [20]. Pigneur et al. reported that of 10 VEOIBD patients with IL10 signalling pathway defects, 8 patients underwent surgery, 5 patients were clinically stable after HSCT, 2 patients died of B-cell lymphoma before HSCT and 1 patient stabilized without treatment [21]. Our group obtained the same results: 5 patients died after emergency surgery because of severe infection and/or malnutrition. After elective enterostomy, only 1 patient died because of sepsis during HSCT. The timing of enterostomy and the age at enterostomy should be considered. The benefits of selective enterostomy were superior to those of emergency enterostomy. Prophylactic enterostomy at an earlier stage may be indicated in some cases. The most common indications for elective surgery include severe perianal diseases, such as strictures and fistulas; severe intestinal infection with persistent diarrhoea and septicaemia; and aggressive disease after medical treatment. The risk of life-threatening infections will increase in cases of delayed or unnecessary operations. Therefore, the timing of surgery is particularly important for these patients. According to our group’s experience and the published literature, if aggravated infection or abdominal distension and intestinal dynamic obstruction occur, an enterostomy should be performed immediately [7, 22].

The management of severe VEOIBD with IL10 gene mutations remains a challenge despite significant advances in medical and surgical therapies [15, 23, 24]. The burden of surgery and postoperative complications in children with IBD should be considered [25, 26]. In our study, although the drug expenditure decreased after enterostomy because of remission, the total expenditure did not decrease significantly because of the additional expenses associated with stoma care. Furthermore, more than 50% of patients experienced complications after colostomy, and the complication rate remained considerably high. Wound infection, wound dehiscence, reoperation, short bowel syndrome, etc. occurred in our patients. Stoma care and routine assessments were important. Stoma construction apart from the primary incision may also reduce complications. Furthermore, stoma closure was another issue for the patients who underwent enterostomy.

Assa et al. reported that for paediatric Crohn’s disease (CD), complicated presentations at diagnosis were associated with an increased risk of surgery (HR = 2.7, *P* < 0.001), and for ulcerative colitis, severe disease at diagnosis was associated with an increased risk of colectomy (HR = 3.5, *P* = 0.002) [27]. Ladd et al. reported that a multivariable analysis indicated that malnutrition corresponded with increased odds of complications in mild and severely malnourished patients (mild OR = 2.1 [*P* = 0.04], severe OR 3.26 [*P* = 0.03]) [9]. In our study, the multivariate logistic regression analysis showed that although there was no significant difference, the CRP level and wPCDAI showed tendencies to be independent risk factors.

Our study had limitations. One limitation was the small sample of enterostomy patients because VEOIBD with an IL10R gene mutation is still a rare disease. Therefore, there was no significant difference in the independent risk factors. Second, increased mortality may have influenced the long-term complication rate for enterostomy. Finally, it was difficult to objectively define the patients’ disease severity because VEOIBD occurred at a very young age.

## Conclusions

Our study demonstrated the benefits of enterostomy in IL10R-mutation VEOIBD patients. The timing, associated complications and related risk factors of enterostomy should be considered.

## Supplementary information

**Additional file 1: Table S1.** The pathogenic/likely pathogenic variants in VEOIBD patients with IL10R gene mutation.

## Data Availability

The datasets used and analysed in the current study are available from the corresponding author upon reasonable request.

## Abbreviations

BMI: Body mass index
CRP: C-reactive protein
FMT: Faecal microbiota transplant
HSCT: Haematopoietic stem cell transplantation
IL10: Interleukin-10
IQR: Interquartile range
SD: Standard deviation
VEOIBD: Very early onset inflammatory bowel disease
WHO: World Health Organization
wPCDAI: weighted paediatric Crohn’s disease activity index
