# Supplementary material for: Complications of enterostomy and related risk factor analysis of very early onset inflammatory bowel disease with interleukin-10 signalling deficiency: a single-centre retrospective analysis
Source: BMC Gastroenterol. 2020 Jan 13;20:8. doi: 10.1186/s12876-020-1160-4 (PMC6958732; doi:10.1186/s12876-020-1160-4)
Supplement: Supplementary file 1 — Additional file 1: Table S1. The pathogenic/likely pathogenic variants in VEOIBD patients with IL10R gene mutation. [file 12876_2020_1160_MOESM1_ESM.docx]

# Table S1: the pathogenic/likely pathogenic variants in VEOIBD patients with IL10R gene mutation

| NO | gene | Site 1 | variants score | Site 2 | variants score |
| --- | --- | --- | --- | --- | --- |
| 1[1] | IL10RA | Exon3: c.299T>G (p.V100G) | LP | Exon3: c.301C>T (p.R101W) | P |
| 2[1] | IL10RA | Exon4: c.537G>A (p.T179T) | P | Exon3: c.301C>T (p.R101W) | P |
| 3[1] | IL10RA | Exon3: c.301C>T (p.R101W) | P | Exon3: c.301C>T (p.R101W) | P |
| 4[1] | IL10RA | Exon3: c.301C>T (p.R101W) | P | Exon3: c.301C>T (p.R101W) | P |
| 5[1] | IL10RA | Exon3: c.299T>G (p.V100G) | LP | Exon4: c.537G>A (p.T179T) | P |
| 6[1] | IL10RA | Exon4: c.537G>A (p.T179T) | P | Exon3: c.301C>T (p.R101W) | P |
| 7[1] | IL10RA | Exon6:C.746delC | P | Exon3: c.301 C>T (p.R101W) | P |
| 8[1] | IL10RA | Exon3: c.301C>T (p.R101W) | P | Exon3: c.301C>T (p.R101W) | P |
| 9[1] | IL10RA | Exon3: c.301C>T (p.R101W) | P | Exon5: c.569T>G(p.F190>C) | LP |
| 10[1] | IL10RA | Exon2: c.99G>A (p.W33X) | P | Exon3: c.301C>T (p.R101W) | P |
| 11[2] | IL10RA | Exon5: c.634C>T (p.R212X) | P | Exon4: c.537G> A (p.T179T) | P |
| 12[3] | IL10RA | Exon3: c.301C>T (p.R101W) | P | Exon4: c.537G>A (p.T179T) | P |
| 13[3] | IL10RA | Exon3: c.350G>A (p.R117H) | LP | Exon4: c.493C>T (p.R165X) | P |
| 14[3] | IL10RA | Exon3: c.301C>T (p.R101W) | P | Exon3: c.301C>T (p.R101W) | P |
| 15 | IL10RA | Exon4: c.537G>A (p.T179T) | P | Exon2: c.99G>A (p.W33X) | P |
| 16 | IL10RA | Exon3: c.301C>T (p.R101W) | P | Exon4: c.537G>A (p.T179T) | P |
| 17 | IL10RA | Exon3: c.299T>G (p.V100G); | LP | Exon3: c.301C>T (p.R101W) | P |
| 18 | IL10RA | Exon3: c.301C>T (p.R101W) | P | Exon4: c.493C>T (p.R165X) | P |
| 19 | IL10RA | Exon3: c.301C>T (p.R101W) | P | Exon4: c.537G>A (p.T179T) | P |
| 20 | IL10RA | Exon3: c.301C>T (p.R101W) | P | Exon4: c.537G>A (p.T179T) | P |
| 21 | IL10RA | Exon3: c.301C>T (p.R101W) | P | Exon4: c.537G>A (p.T179T) | P |
| 22 | IL10RB | Exon4: c.612G>C (p.W204C) | VUS | Exon4: c.612G>C (p.W204C) | VUS |

LP: likely pathogenic, pathogenic; VUS: variants of unknown significance

Reference

1. Huang Z, Peng K, Li X, Zhao R, You J, Cheng X, Wang Z, Wang Y, Wu B, Wang H *et al*: **Mutations in Interleukin-10 Receptor and Clinical Phenotypes in Patients with Very Early Onset Inflammatory Bowel Disease: A Chinese VEO-IBD Collaboration Group Survey**. *Inflammatory bowel diseases* 2017, **23**(4):578-590.

2. Ye Z, Zhou Y, Huang Y, Wang Y, Lu J, Tang Z, Miao S, Dong K, Jiang Z: **Phenotype and Management of Infantile-onset Inflammatory Bowel Disease: Experience from a Tertiary Care Center in China**. *Inflammatory bowel diseases* 2017, **23**(12):2154-2164.

3. Zheng C, Huang Y, Hu W, Shi J, Ye Z, Qian X, Huang Z, Xue A, Wang Y, Lu J *et al*: **Phenotypic Characterization of Very Early-Onset Inflammatory Bowel Disease with Interleukin-10 Signaling Deficiency: Based on a Large Cohort Study**. *Inflammatory bowel diseases* 2019, **25**(4):756-766.
